# Extra-classical receptive field effects measured in striate cortex with fMRI

**DOI:** 10.1016/j.neuroimage.2006.10.017

**Published:** 2007-02-01

**Authors:** L.M. Harrison, K.E. Stephan, G. Rees, K.J. Friston

**Affiliations:** a The Wellcome Dept. of Imaging Neuroscience, Institute of Neurology, UCL, 12 Queen Square, London WC1N 3BG, UK; b Institute of Cognitive Neurology, University College London, UK

## Abstract

The aim of this study was to measure the contextual influence of globally coherent motion on visual cortical responses using functional magnetic resonance imaging. Our motivation was to test a prediction from representational theories of perception (*i.e.* predictive coding) that primary visual responses should be suppressed by top-down influences during coherent motion. We used a sparse stimulus array such that each element could not fall within the same classical receptive field of primary visual cortex neurons (*i.e.* precluding lateral interactions within V1). This enabled us to attribute differences, in striate cortex responses, to extra-classical receptive field effects mediated by backward connections. In accord with theoretical predictions we were able to demonstrate suppression of striate cortex activations to coherent relative to incoherent motion. These results suggest that suppression of primary visual cortex responses to coherent motion reflect extra-classical effects mediated by backward connections.

## Introduction

**Fig. 1:**
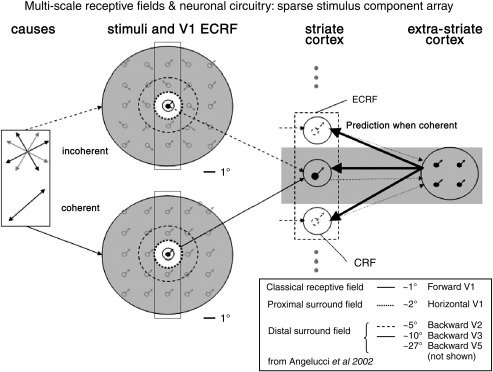
Multi-scale receptive fields and neuronal networks. Causes of either coherent or incoherent motion (left) generate a stimulus, which provides data to the primary sensory cortex. A neuron processing the central component receives input directly from the stimulus (feed-forward) and additional information from backward connections. The ECRF is composed of a proximal and a distal surround field whose spatial extent is consistent with horizontal and backward sources of influence respectively (Angelucci et al., 2002). By using a sparse array a bias is placed on backward, over horizontal, pathways to provide contextual guidance, as only one stimulus component can fall within the proximal surround field of a V1 neuron (dimensions reported in Angelucci et al., 2002). Abbreviations: V1 = striate cortex, (E)CRF = (extra) classical receptive field.

Neurobiological theories of representational learning and perception usually invoke a cortical hierarchy with forward and backward connections (Lee and Mumford, 2003; Friston, 2003; Mesulam, 1998; Rao and Ballard, 1999; Olshausen and Field, 1996). Hierarchical architectures are based on evidence from anatomy and physiology (Felleman and Van Essen, 1991; Angelucci et al., 2002; Albright and Stoner, 2002), developments in theoretical neuroscience *e.g.* neural networks (Dayan and Abbott, 2001) and statistics, *e.g.* combining probability and graph theory to form graphical models (Jordan, 1999). Hierarchical Bayesian theories of cortical inference (*e.g.* predictive coding; Friston, 2003; Rao and Ballard, 1999) treat the visual system as a hierarchy of cortical areas that tune themselves to features, or patterns, within visual data at multiple scales. Critically, each level depends on both top-down and bottom-up information, so that the ensuing activity of any level reflects the integration of these two influences. The aim of this study was to see whether responses in visual regions processing local visual motion are sensitive to the global context of motion even when, from a local perspective, the stimuli are identical (see Fig. 1 and below). In particular, as has been postulated theoretically, the detection of structural regularities should lead to *decreased* activity in V1 because these regularities enable the hierarchy to predict incoming sensory data more efficiently (*i.e.* with less prediction error) (Rao and Ballard, 1999; Koch and Poggio, 1999).

Angelucci and colleagues (Angelucci and Bullier, 2003; Levitt and Lund, 2002) extended previous work (Nelson and Frost, 1978; Blakemore and Tobin, 1972; Sceniak et al., 1999; Sengpiel et al., 1997) on receptive field (RF) sizes in macaque primary visual cortex by measuring the spatial extent of orientation-specific surround suppression of parafoveal V1 cells in the macaque and compared their spatial scales with the visuotopic extent of forward, horizontal (or lateral) connections within V1 and backward connections from extra-striate cortex. It has long been known that the classical RF has a suppressive surround field whose more central parts are modulated by high-contrast stimuli and whose more peripheral parts are modulated by low-contrast stimuli. Angelucci et al. found that the spatial extent of the high-contrast summation field (hsRF)^1^ is consistent with that of terminations of forward connections, and the spatial extent of low-contrast summation field (lsRF) is consistent with that of horizontal connections^2^ (Angelucci and Sainsbury, 2006). The spatial extent and conduction velocities of these connections, however, cannot account for the spatial scale and timing of the full surround suppression field^3^. Instead, Angelucci et al. concluded that these effects are commensurate with the visuotopic extent^4^ and conduction velocities^5^ of backward projections from extra-striate cortex (Girard et al., 2001). In Angelucci and Bullier (2003) they refer to the region between hsRF and lsRF as the ‘proximal surround’ and beyond this as the ‘distal surround’. The surround field can then be conveniently thought of as containing ‘proximal’ and ‘distal’ components that depend on horizontal connections and inter-areal backward connections respectively. These comprise the extra-classical receptive field (ECRF). A schematic of these ideas is shown in Fig. 1. Contextual effects in V1 may therefore depend on afferents from V2 or higher regions.

**Fig. 2:**
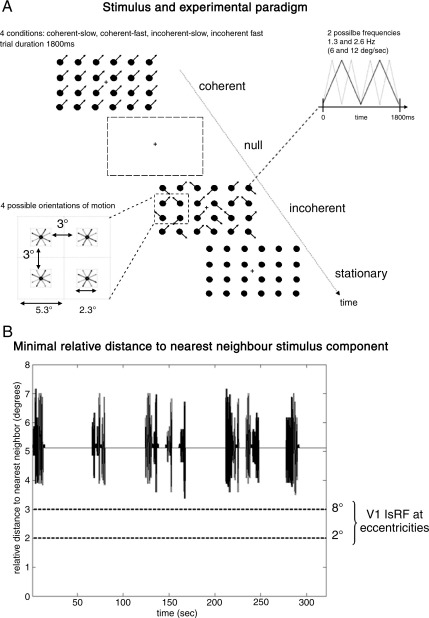
Stimulus and experimental paradigm. A) Lower left panel: each component moved such that all components were a minimum of 3° apart throughout both coherent and incoherent motion trials. The motion of each component was constrained to one of 4 orientations. A) Upper right: motion stimuli were presented at two different speeds [frequencies] (6 or 12°/s [1.3 or 2.6 Hz]). (A) Center figure: coherent [incoherent] motion stimuli were interleaved with ‘null’ and ‘stationary’ trials. (B) Relative distance between a stimulus component and its nearest neighbor over one session. This is constant for coherent motion (5.3°) and varies for incoherent. The minimal distance during incoherent motion is approximately 3.5°. V1 lsRF sizes at retinal eccentricities 2° and 8° (Angelucci et al., 2002) are shown to illustrate that from a local perspective coherent and incoherent stimuli are identical.

The key feature of the current study is that we used a *sparse* stimulus array motivated by the ECRF dimensions in V1 macaque reported in Angelucci et al. (2002). Given a mean proximal surround field of ∼ 2.3° (Angelucci et al., 2002) our stimulus was designed such that all components were a minimal distance of 3° apart at all times. See Fig. 2A (lower left panel) for stimulus dimensions and 2b for a plot of relative distance to the nearest neighbor stimulus component. Under the assumption that human receptive fields share similar spatial scales to macaque this enabled us to attribute the effects of coherency to extra-classical effects mediated by backward connections.

## Methods and materials

We scanned 12 normal subjects using a 3 Tesla scanner to measure the Blood Oxygenation Level Dependent (BOLD) responses evoked by a sparse array (4 × 6) of circular components (each subtending 0.5°) oscillating either coherently or incoherently at 2 different frequencies. Subjects were instructed to fixate a central cross and their eye movements were recorded. The individual stimulus components oscillated about a fixed point along 4 possible orientations. All components were a minimal distance of 3° apart so that the global movement of the array, *i.e.* either coherent or incoherent, could not be discerned by observation of a single component. Each subject performed 1024 trials of 1800 ms, including 256 null trials. Four stimulus conditions were presented; coherent-slow, coherent-fast, incoherent-slow or incoherent-fast. This factorial design allowed us to measure the difference in BOLD responses between coherent and incoherently moving components, at two different frequencies. See Fig. 2 for the stimulus and paradigm.

### Subjects

Written informed consent was obtained from 12 right-handed subjects (8 males; age range 22–35 years; mean age 27) with normal eyesight and no neurological impairments. Ethics approval was obtained from the joint ethics committee of the Institute of Neurology, University College London and National Hospital of Neurology and Neurosurgery, London.

### Visual stimuli

Single trials were presented for 1800 ms using a Liquid Crystal Display projector and reflected from a small mirror above the subject's head. The screen was divided into a 4 × 6 mesh such that each element measured 160 × 160 pixels (∼ 5.3 × 5.3°) and contained a smaller square 70 × 70 pixel (∼ 2.3 × 2.3°) at its center (see lower left of Fig. 2A). Each stimulus component was a filled white circle of diameter 16 pixels (∼ 0.5°) on a black background whose initial position was at the center of each square. Movement of each circle was along one of four orientations (± 26.5 ± 90° relative to vertical) of equal length with one of two possible initial directions separated by 180°. The components traveled at a constant speed of either 6 or 12°/s and, as each trial lasted 1800 ms, had frequencies of 1.3 or 2.6 Hz. Each circle remained within the boundary of the lesser square so that the minimal distance between components was 90 pixels (∼ 3°) at all times. Minimum and maximum radial eccentricities were approximately 2.3° and 16.3° respectively. During coherent motion an orientation was sampled from a uniform distribution along which all stimulus components moved at the same frequency throughout a trial. Conversely during incoherent presentations orientations were sampled for each component with the constraint that no more than one immediate neighbor had the same direction of motion and orientation. This was to exclude the random selection of regionally coherent motion. Each component moved at the same frequency throughout a trial. Subjects were instructed to fixate on a central white cross during all trials.

The experiment consisted of four sessions each containing 256 trials (duration ∼ 8 min/session). Of these 64 were ‘null’ events where the fixation cross was presented alone. The 192 remaining events were trials of which 64 were ‘stationary’ where all stimulus components remained at their initial positions and 128 trials were divided into 32 trails for each of the four conditions; coherent-slow, coherent-fast, incoherent-slow and incoherent-fast. This represents a factorial event-related design, in which two factors were manipulated orthogonally: the global context of motion (coherent or incoherent) and the speed [frequency] of moving stimulus components (6 or 12°/s [1.3 or 2.6 Hz]). Trials from each condition were then interleaved and counterbalanced with ‘stationary’ and ‘null’ events to make blocks containing 16 events. These blocks were then ordered stochastically to ensure independence among conditions. The experiment was conducted in short sessions (∼ 8 min) and included an incidental task that maintained attentional set: subjects were asked to respond when the stimulus components were red. There were 12 task events selected at random in each session, which subjects responded with a button press using the right index finger.

### Data acquisition

#### Eye tracking

To measure deviations from fixation, eye movements were recorded during each session using ASL transfer with remote optics. We recorded *x* and *y* coordinates of eye movement, pupil diameter and the onset/offset of each experimental event.

#### Functional imaging

A 3T Siemens ALLEGRA system was used to acquire T1-weighted anatomical images and gradient-echo echo-planar T2*-weighted MRI image volumes with Blood Oxygenation Level Dependent (BOLD) contrast. A total of 960 volumes were acquired per subject plus 6 initial ‘dummy’ volumes to allow for T1 equilibration effects. Volumes were acquired continuously every 2506 ms. Each volume comprised 33 3.3 mm axial slices, with an in-plane resolution of 3 × 3 mm, positioned to cover the entire cerebrum.

### Data processing

#### Eye tracking

The mean trial displacement was calculated using robust averaging (using a mixture of Gaussian models) to accommodate artifacts *e.g.* eye blinks. Trial averages were entered into a 2 level hierarchical model to measure random effects (Kiebel et al., 2003) over subjects and quantify differences between eye movement amplitudes during coherent and incoherent trials. Eye movement data were also analyzed in the frequency domain. Time-series of each trial were transformed (0–8 Hz in 0.5 Hz increments) and concatenated into a general linear model to test for differences between coherent and incoherent motion stimuli.

#### Functional imaging

The imaging time series were realigned, unwarped, normalized into a standard anatomical space (Ashburner and Friston, 1997) and smoothed with a Gaussian kernel of 6 mm full width half maximum. The data were analyzed using Statistical Parametric Mapping (SPM2), employing an event-related model (Friston, 2004) and a two-stage random effects procedure. Trials were modeled by convolving ‘delta’ functions, for each trial, with a canonical hemodynamic response function (HRF). These explanatory variables were used as regressors in a general linear model (Friston, 2004) and included the four types of motion (coherent-slow, coherent-fast, incoherent-slow and incoherent-fast), stationary and task events. For the random effects analysis subject-specific contrast of the main effect of coherency were entered into a one-sample *t*-test. We chose a significance level of *p* < 0.01 whole-brain corrected at the cluster-level (with a standard *p* < 0.001 cut-off at the voxel-level) and performed additional small-volume corrections for two areas, right extra-striate cortex and bilateral posterior cingulate gyrus (pCG), that achieved a significance level of *p* < 0.05 whole-brain uncorrected at the cluster level and were implied by previous studies on coherent and incoherent motion (Dougherty et al., 2003).

## Results

### Eye tracking

**Fig. 3:**
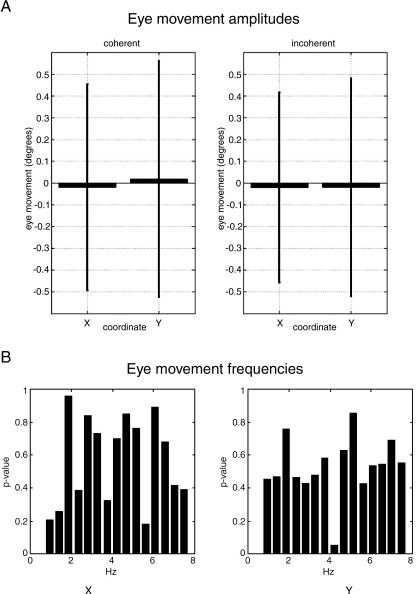
(A) Eye movement amplitudes. Mean and standard deviation of eye movement (degrees) from fixation measured in *x* and *y* coordinates during coherent (left) and incoherent motion. Their respective means (std) were [− 0.02 (0.47), 0.02 (0.54)] and [− 0.02 (0.43), 0.02 (0.50)] degrees. *P*-values above a Bonferroni corrected significance level confirm the visibly clear equivalence over conditions. (B) Eye movement frequencies. *P*-values comparing the frequency profile of eye movements during coherent and incoherent motion in *x* (left panel) and *y* directions. There were no significant differences between conditions.

Mean and standard deviation of eye movement amplitudes during coherent (*x*, *y* coordinates [− 0.02 (0.47), 0.02 (0.54)] degrees, standard deviation in parentheses) and incoherent motion ([− 0.02 (0.43), 0.02 (0.50)] degrees) are shown in Fig. 3A. There were no significant differences between conditions of coherent and incoherent motion (*x*, *y* coordinate *F*-statistic [1.3, 1.5], *p*-value [0.1, 0.07] (uncorrected). No differences in the spectral profile of eye movements were detected between conditions. The *p*-values (uncorrected) of a contrast comparing coherent and incoherent motion trials are shown in Fig. 3B.

### Functional imaging

**Fig. 4:**
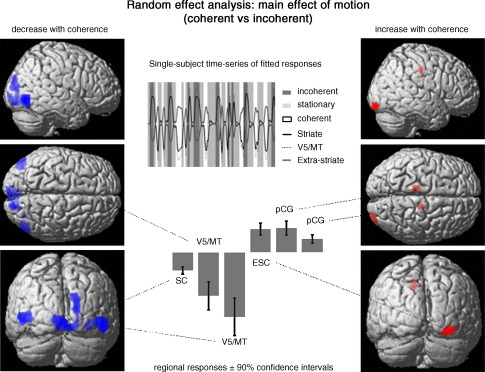
Random effects (between subject) analysis of the main effect of motion context (coherent versus incoherent motion). Left panel) Regions that responded less to coherent motion. Results are displayed at a threshold of *p* < 0.01 whole-brain cluster-level corrected (with *p* < 0.001 voxel-level cut-off). (Right panel) Regions that showed enhanced responses to coherent motion. Results are displayed at a threshold of *p* < 0.01 small-volume (20 mm radius) correction (with *p* < 0.001 voxel-level cut-off). Parameter (*i.e.* visually evoked response) estimates of the general linear model are shown in the lower central graph. Top center compares fitted data from one subject over one session and illustrates the opposing responses measured in striate, extra-striate cortex and human V5/MT. Abbreviations: SC = striate cortex, ESC = extra-striate cortex, V5 = human V5/MT, pCG = posterior cingulate gyrus.

Results from a random effects (*i.e.* between subject) analysis of the main effect of coherent relative to incoherent motion are summarized in Tables 1 and 2. Fig. 4 shows the Statistical Parametric Maps (SPMs), projected on to a 3D canonical image of the cortical surface.

The main effect of coherent relative to incoherent motion was a bilateral and symmetric pattern of responses in occipital and parietal lobes. On the left of Fig. 4 the SPMs demonstrate a strong effect of coherent motion with maxima of decreased visually evoked response in striate cortex and human V5/MT. The right figure shows increased activations in the right extra-striate cortex adjacent to striate cortex and bilateral posterior cingulate gyrus (pCG) during coherent motion. In addition we did a small volume search of these 2 regions based on Talairach coordinates reported in (Dougherty et al., 2003) and (Law et al., 1997). The bottom central panel shows estimated responses in these regions (with 90% confidence intervals). The top central panel is a typical example of the fitted BOLD response of one subject during the experiment. These results illustrate the opposite responses of the three regions.

**Fig. 5:**
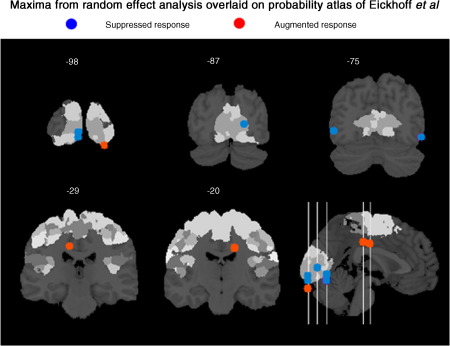
The maxima from the random effects analysis, for the main effect of motion context (shown in Fig. 4), are overlaid on the probability atlas of Eickhoff et al. (2005).

For anatomical designation, the positions of maximum effects from the random effects analysis are overlaid on coronal sections of the probability atlas of Eickhoff et al. (2005) in Fig. 5. These are indicated with either a blue (decreased response to coherent motion) or red sphere (increased response). The probability atlas delineates, among others, Brodmann areas 17 and 18 of the occipital cortex.

## Discussion

The aim of the current study was to measure the differential responses of occipital cortex to a *sparse* array of globally coherent [incoherent] moving stimulus components using fMRI. Our measurements clearly demonstrate that responses to coherent [versus incoherent] motion engage regions of the occipital and parietal lobes. In particular the maxima of reduced responses to coherent stimuli were seen in striate cortex and human V5/MT, while an enhanced response was measured in extra-striate cortex and the posterior cingulate gyri. Critically, the relative suppression of striate cortex was seen even though each classical RF of striate cortex was only affected by a single moving dot of our stimulus. This suggests that suppression, in the context of coherent and thus predictable stimuli, is mediated by backward connections from higher areas.

Previous PET and fMRI studies of occipital responses to coherent motion that used stimulus components (as opposed to plaids; Adelson and Movshon, 1982) to depict globally coherent [incoherent] motion include McKeefry et al. (1997), Rees et al. (2000) and Braddick et al. (2001). McKeefry et al. used ∼ 100 square components, each subtending 0.66°, randomly distributed, with occasional overlap (*c.f*., the 24 components with a minimal spacing of 3° used in our study). They reported relative suppression in V1, V2, V3 and V5/MT to coherent motion when compared to incoherent. They did not detect significant increases due to coherency, which they expected to observe in V5/MT. We recorded suppression of a large cluster (see Table 1) with maxima within the striate as well as in the region of human V5/MT. Given the extent of the occipital response it is likely to extend beyond striate to extra-striate cortex. In addition to these findings we have identified an extra-striate region, distinct from V5/MT and adjacent to striate cortex, whose response increased. This is important as representational theories predict concurrent suppression and enhanced responses of sub and supra-ordinate regions respectively. The probability atlas locates the maximum of this activation on the border between V1 and V2. This is a population effect, *i.e.* random effects analysis, and as such does not provide the spatial precision of single-subject retinotopic mapping. However, given a population measure, the probability atlas is currently the optimal way to quantify uncertainty as to a specific regional designation. The robustness of this approach has been shown in comparative studies between the cytoarchitectonic probability maps of (i) V1 and V2 compared to their retinotopic delineation (Wohlschlager et al., 2005) and (ii) V5/MT with fMRI responses (Wilms et al., 2005).

Braddick et al. used a stimulus with component density over 100 times that of McKeefry et al. They reported relative decreases to coherent motion in V1 and increases throughout V2, V3 and human V5/MT. In addition, Rees et al. used a high component density of ∼ 20 dots/° (each dot subtending ∼ 2 arc min) within circular apertures of 2° radius centered 4° symmetrically to the left and right of a fixation cross. By varying the percentage of coherently moving dots they were able to show a positive linear relationship between percentage coherency and responses of V2 and human V5/MT.

The density we employed was informed by anatomical and physiological measurements of the visuotopic extent of horizontal connections in macaque V1. Assuming similar characteristics in human cortex (Smith et al., 2001) this afforded a principled choice of component density to investigate putative top-down influences of extra-striate on striate cortex responses. By using a sparse stimulus array we introduced a bias away from horizontal connections among V1 neurons encoding individual stimulus components and towards feedback connections from extra-striate cortex that represent a wider spatial context. Our stimulus ensured that at any given time the RF of any individual V1 neuron would only be affected by a single dot of the display. This holds true even when taking into account that there is an approximately linear increase in mean parafoveal receptive field (RF) width of V1 neurons with eccentricity. Average RF width in V1 increases from ∼ 0.25° in fovea to ∼ 1.3° at 20° eccentricity (Hubel and Wiesel, 1974), and the minimum and maximum radial eccentricity of the dots in our stimuli were 2.3° and 16.3°, respectively. In addition, constraining motion to four orientations meant that coherent and incoherent stimuli were indistinguishable when observing any component in isolation. Overall, this meant the stimulus was well controlled for V1 RF sizes (see Fig. 2B) and that any contextual dependence of V1 responses must be mediated by afferents other than geniculate input or horizontal interactions, *e.g.* a top-down source of the sort described in accounts of ECRFs (Angelucci and Bullier, 2003). In short, the differential V1 response for coherent versus incoherent motion is most easily explained in terms of the influence of backward projections from higher areas whose receptive fields span a wider spatial field and exhibit statistical dependence. While our stimuli were designed with regard to V1 RF sizes, they were not as well controlled for higher visual areas with larger RFs, *e.g.* extrastriate areas and V5/MT. The findings related to these areas are therefore more difficult to interpret.

The decrease in V5/MT responses during coherent motion is interesting given previous studies discussed above. Furthermore, studies in macaque (Recanzone et al., 1997) and human subjects (Huk and Heeger, 2002; Castelo-Branco et al., 2002) show selective V5/MT responses to component motion (where a surface is perceived to move beneath another, transparent, surface) *and* pattern motion (where the same stimulus is perceived to be one surface moving coherently in one direction). In addition, in a related study, Murray et al. (2002) measured relative suppression in V1 and V5/MT to widely separated moving stimuli (experiment 2 in (Murray et al., 2002)) in the context of an occluder moving over a coloured collinear object. Given these results, the characterization of V5/MT responses is not as clear-cut as V1. The decrease in V5/MT responses to the stimulus used in this study has several possible explanations. These rest on forward inputs to V5/MT via routes that bypass V1 (Sincich et al., 2004), backward suppression from regions supra-ordinate to V5/MT and horizontal suppression within V5/MT. It is interesting to note that in one study MT inactivation by cooling reduced surround suppression in V1 to low salience stimuli, while inactivation of V2 had no effect on V1 surround responses (Bullier et al., 2001). This effect was much less for middle and high salience stimuli. We observed a *relative* decrease in human V5/MT and V1 response to high salience coherent compared to incoherent motion stimuli. A direct comparison of the two studies would suggest reduced center-surround effects in V1 associated with decreased MT activity, which would be inconsistent with our current interpretation. However, a direct comparison is not possible due to salience of the stimulus and the difference between absolute MT inactivation and relative reduction in response, which may not necessarily lead to reduced coupling of center-surround mechanisms.

Our results suggest that V1 and V5/MT have similar response profiles, which are consistent with a low-level in the visual hierarchy. Although V5 is usually thought of as a relatively high-level area, direct inputs from LGN might confer some paradoxical low-level attributes. Related arguments have been made in the context of blindsight (Stoerig and Cowey, 1997), where subjects maintain a degree of visual awareness despite destruction of primary sensory cortex. An explanation is based on connections from LGN to V5 that bypass V1. These comprise about 10% of the geniculate projections in the macaque monkey (Sincich et al., 2004). Latency studies reveal visual signals in macaque V5/MT arrive only a few milliseconds after or sometimes even before V1 (Raiguel et al., 1989; Schmolesky et al., 1998). Trans-cranial stimulation in humans (Beckers and Zeki, 1995) measured the termination of motion perception at shorter times when V5/MT was stimulated compared to V1. These studies suggest that there is an alternative and faster route to V5/MT than via V1. If V5/MT receives sensory input at a similar stage of processing as V1 then one might expect V5/MT and V1 to respond similarly in some contexts. That is, V5/MT activity may reflect prediction error that is suppressed by supra-ordinate regions during a predictable stimulus. Macaque V5/MT receptive fields are larger than V1 (Mikami et al., 1986) (∼ 5° near the fovea and increase to ∼ 30° at 30° eccentricity) with 10–15° overlap with the ipsilateral hemisphere (Raiguel et al., 1995, 1997). This means that even a sparse stimulus array will not have the same effect on horizontal interactions as expected in V1. Given this, the proximal surround field due to horizontal interactions within V5/MT (Raiguel et al., 1995) could account for a suppressed response.

It is interesting that we measured increased responses bilaterally in the posterior cingulate gyri during coherent motion, which suggests a possible source of top-down prediction. Activity in these cells, may exert a suppressive influence over the distal surround of MT neurons.^6^ The literature with regards to connections from posterior cingulate cortex to MT is sparse. Although no direct connection has yet been reported, there is an indirect connection via areas in the superior temporal sulcus. In particular, the posterior part of area TPO, which is located next to MT, receives a strong input from posterior cingulate cortex (Parvizi et al., 2006) and projects itself to area V5/MT (Seltzer and Pandya, 1989).

The extra-striate cortical response was significantly enhanced by coherent motion and may represent a source of prediction that suppressed striate responses. V2 is involved in the perception of dynamic form as V1 projections to V3 and V5 pass via the thick stripes of V2 (Zeki, 1993). Angelucci and Bullier (2003) present evidence that the ECRF of V1 cells depend on horizontal V1 connections (~ 2°) and feedback coupling from V2 (∼ 5°), V3 (∼ 10°) and V5 (∼ 27°). This is important for our experiment because V2 is the first level of hierarchical processing where peripheral units receive sensory inputs from more than one of our stimulus elements (*i.e.* V2 is the first level with access to contextual information inherent in coherent motion). We illustrate this in Fig. 2B, which shows the relative distance between nearest neighbor stimulus components along with the low-contrast summation field (lsRF) sizes of a typical V1 neuron (∼ 2° and 3°) at 2° and 8° retinal eccentricity (Angelucci et al., 2002). Given V2 RF sizes are approximately twice those of V1, lsRF are approximately 4° and 6° at eccentricities of 2° and 8°. As the range of radial eccentricity of our stimulus was ∼ 2–16°, V2 cells were exposed to a stimulus similar to V1 within the parafoveal region, however, beyond this region it is likely that V2 cells were exposed to two stimulus components. Note that we did not adjust the inter-dot spacing in the stimulus to account for changes in RF size with eccentricity. Restricting our stimulus to a more limited field of view, or adjusting the inter-dot spacing are potential manipulations, which we will pursue in subsequent work. This interpretation suggests, however, that a further study where inter-dot spacing increases with eccentricity and that explicitly changes the spacing is required. This would provide a well-controlled method to investigate changes in striate, extra-striate and human V5/MT responses as a function of the relative distance among neighboring components, and to see whether the extrastriate responses we observed depends on inter-dot distance and whether V5/MT activates with larger inter-dot distances.

The local contextual information inherent in the dynamic form of globally coherent motion could contribute to representations of global movement associated with V5/MT responses. It is interesting to note that computational models of Weiss et al. (1998) and Sajda and Baek (2004), which are based on notions from hierarchical Bayesian modelling, depend crucially on the integration of uncertainties in form and motion to deal with the inevitable ambiguity that arises from visual inputs. It is also of interest that there are virtually no [extra-foveal] inter-hemispheric connections at the level of V1 yet there are a large number of commissural fibers between V2 (Aboitiz, 1992; Van Essen et al., 1982). If this region is supra-ordinate to V1 in the context of coherent motion, then rapid access to inter-hemispheric computations may be essential.

Our results accord closely with those of Murray et al. (2002). These authors also tested a hypothesis derived from predictive coding theories, but in the domain of formal information and object recognition. They used fMRI to measure activity throughout the visual cortex during presentation of stimuli that were either grouped into objects or randomly arranged. They found significant increases of activity in lateral occipital complex (LOC) and reduced responses in V1 when elements formed coherent shapes. It is interesting that they too measured relative suppression in V1 and V5/MT to the coherent movement of widely separated objects (see experiment 2 in (Murray et al., 2002)). As in our study, these results conform to predictions from hierarchical Bayesian theories of predictive coding that postulate a decrease in activity when incoming data from lower levels match the predictions from higher levels in the hierarchy. In our study, we found corresponding decreased responses in striate cortex and human V5/MT during coherent motion, while increased activity was measured in V2.

## Conclusion

Our results have a natural interpretation in terms of inferential theories of perception, in particular predictive coding, where representations of global percepts influence local processing at subordinate levels. These theories rest on recognition in hierarchical networks, where backward connections facilitate perception through the process of prediction. This study lends additional credibility to the use of fMRI in testing predictions from computational models of representational learning and perception. Although we have only tested a basic prediction from this theory, our results suggest that predictive coding models may be a useful metaphor for interpreting functional activations and could form the basis for observation models in neuroimaging (Friston, 2005).

## Figures and Tables

**Table 1:** Main effect of motion (coherent versus incoherent)

Lobe	Region	Cluster extent	Cluster level *p*_corected_	*Z* score	Coordinates		
*x*	*y*	*z*
Occipital	Striate cortex	686	0.000	4.94	− 2	− 98	0
0.000	4.44	0	− 92	− 6
0.000	4.12	18	− 86	10
Left V5/MT	191	0.001	3.98	− 50	− 74	2
Right V5/MT	345	0.000	4.19	52	− 74	− 6

Coordinates, *Z* scores and corrected *p*-values of regions that showed relative *suppression* in response to coherent motion.

**Table 2:** Main effect of motion (coherent versus incoherent)

Lobe	Region	Cluster extent	Cluster level *p*_corrected_	*Z* score	Coordinates		
*x*	*y*	*z*
Occipital	Right extra-striate cortex	64	0.007 (20 mm svc)^a^	4.45	28	− 98	− 16
Parietal	Left pCG	75	0.003 (20 mm svc)^b^	4.19	− 12	− 28	42
	Right pCG	50	0.018 (20 mm svc)^b^	4.07	16	− 20	40

Coordinates, *Z* scores and corrected *p*-values of regions that showed relatively *enhanced* response to coherent motion. Abbreviations: pCG = posterior cingulate gyrus, V5 = human V5/MT, svc = small volume correction.

a Centered on [25, − 80, 9] from Dougherty et al. (2003).

b Centered on [0, − 24, 48] (see Law et al., 1997 for comparison).

## Footnotes

1 Same abbreviations used in Angelucci et al. (2002).

2 Mean hsRF ∼ 1° and lsRF ∼ 2.3° for neurons within 2–8° retinal eccentricity (see Fig. 2c; Angelucci et al., 2002).

3 Modulatory surround field can be beyond 13°.

4 Visuotopic extent of V1 horizontal connections is ∼ 2°, while feedback from V2, V3 and macaque V5/MT is ∼ 5°, ∼ 10° and ∼ 27° respectively (Angelucci et al., 2002).

5 Conduction velocities of horizontal connections are 10 times slower than forward and backward connections (Angelucci and Bullier, 2003; Angelucci and Sainsbury, 2006).

6 A PET study by Law et al. (1997) measured a large region of activation in the caudal cingulate/supplementary eye fields centered at Talairach coordinates [0, − 8, 48] in response to suppression of eye saccades when observing a moving stimulus. Their activation is anterior to the regions we have labelled posterior cingulate gyri (small volume correction centered at [0, − 24, 48], see Table 2) but may subsume it.
